# The Care of the Baby

**Published:** 1924-04

**Authors:** 


### THE CARE OF THE BABY.

Viscount Astor had a satisfactory year's progress to report at the Annual Meeting of National Baby Week Council, which took place at Carnegie House on March 12. There was, however, he said, a danger that they might become apathetic and satisfied with what had been accomplished. The reduction of the infantile mortality to 69 was most encouraging, but there was much to do still in that direction. The average for Cambridge, for instance, was only 38, but at Oldham it was 112. Again, 80 to 90 per cent. of children were born healthy, but at the age of five 35 to 40 per cent. had acquired some physical defect. That had got to be remedied and the root causes attacked. It was their job to make the nation say what the Queen herself had said in a recent message to the Council, "that her interest in the movement was unabated."

### Conference on Birth Control.

After the reading of the Annual Report by Dr. Eric Pritchard, Chairman of the Executive Committee, and the election of officers, a conference was held on "The Size of the Family and its Economic Circumstances in relation to the Welfare of Children under Five." This practically resolved itself into a debate on Birth Control, in which the more conservative standpoint was taken by Principal Garvie and Lady Nott-Bower. Dr. Elizabeth Sloan-Chessar urged that instruction on the use of contraceptives might be given to mothers when necessary at infant welfare centres and that the Ministry of Health should remove its taboo on the subject. Breezy speeches were contributed by Mr. E. B. Turner, F.R.C.S., and the Medical Officer of Health for Deptford.

---

### An X-Ray Victim.

**Efforts are being made to recognise more adequately the work done by Mr. Ernest Harnack, a former X-Ray operator of the London Hospital, who, like his successor Mr. Blackwell, lost both his hands. Mr. Harnack receives a pension of £250 from the hospital and it is thought possible that the Carnegie Hero Fund might consider his case. For a long time after Mr. Harnack was severely affected he stuck to his work. His sufferings were terrible. In co-operation with Messrs. Dean Mr. Harnack invented the lead glass screen which protects present-day operators, but this discovery was too late to save himself.**

